# 

**Published:** 1873-01

**Authors:** 


### Contents of January Number.

### ORIGINAL DEPARTMENT.

ART. I. Case of Spinal Irritation. By S. Weed, M. D., Clyde, N. Y., . . . 201
ART. II. Hysteria with Fibroid Tumor of Uterus. By Benj. A. Fordyce, M. D., Union Springs, N. Y., . . . . . . . . 203
ART. III. Medical Society of the County of Albany, Semi-Monthly Meetings, Dec. 17, 1872 and Jan. 8, 1873. Reported by F. C. Curtis, M. D., Secretary, . . . . . . . . . . 208

Correspondence, . . . . . . . . . . . . . . 218

### MISCELLANEOUS.

Clinical Notes on the Electric Cautery in Uterine Surgery. By J. Byrne, M. D. 220

### EDITORIAL DEPARTMENT.

Erie County Medical Society, . . . . . . . . . . . 236
Meeting of New York State Medical Society, . . . . . . . 238
Vaccine Virus, . . . . . . . . . . . . . . 238
The Magazines, . . . . . . . . . . . . . . 238

BOOKS REVIEWED:

The Microscope and Microscopical Technology. By Heinrich Frey, . . . . . . . . . . . . . 238
The Pathology, Diagnosis and Treatment of Diseases of Women. By Grailey Hewett, M. D., Lond., F. R. C. P., . . 239
A System of Oral Surgery. By James Garretson, M. D., D. D. S., 240

Books and Pamphlets received, . . . . . . . . . . . 240

